# Exploring linkages: addressing the relationship between the climate crisis and HIV prevention with recommendations for emerging pre-exposure prophylaxis programs

**DOI:** 10.3389/frph.2024.1369762

**Published:** 2024-03-26

**Authors:** Katie M. Williams, Adaobi Lisa Olisa, Rose Wilcher

**Affiliations:** ^1^ Science Facilitation, FHI 360, Durham, NC, United States; ^2^ West Africa & Middle East Regional Office, FHI 360, Abuja, Nigeria

**Keywords:** HIV, climate change, prevention, product introduction, PrEP

## Abstract

Short- and long-term impacts of the climate crisis continue to be felt across the public health landscape. Many individuals marginalized by the climate crisis also navigate a higher likelihood of exposure to HIV. By understanding this relationship, we can better position HIV prevention, and pre-exposure prophylaxis (PrEP) programs specifically, to meet user needs in communities experiencing the effects of the climate crisis. In support, we propose four recommendations for mitigating the impact of the climate crisis on those who may benefit from PrEP: (1) leverage existing and emerging research and lived experience to intentionally target and appropriately reach individuals affected by the climate crisis who may need or want PrEP; (2) emphasize the need for more climate-resilient PrEP products within the research and development pipeline; (3) build a continued understanding of the role of the climate crisis-HIV relationship in product introduction through national collaboration; and (4) strengthen the integration of PrEP service delivery and response to intimate partner violence. The PrEP market is set for rapid expansion with the introduction of new prevention methods to enable choice. To be comprehensively responsive to potential PrEP users, we must consider and address how the climate crisis changes not only the environmental landscape, but the prevention ecosystem.

## Introduction

Protracted and acute variables of the climate crisis continue to change the public health landscape and outcomes (1). The specific linkages between HIV and the climate crisis are relatively understudied, primarily because the relationship between the two is largely indirect (2). Yet over the past decade, researchers and advocates from both the health and environment sectors have identified intersections between the two fields and developed a body of work upon which HIV and climate crisis actors can and should build responsive, collaborative programs.

## Understanding the relationship

The climate crisis will continue to affect human migration and displacement, catalyze food scarcity and subsequent insecurity, and destabilize economic opportunities for individuals, all of which may increase vulnerability to HIV (1, 3–5). In addition, both instant and long-term disruption caused by the climate crisis directly affect the lived environment of those with a higher likelihood of HIV exposure (1, 2). Research conducted over the last ten years connects higher rainfall with more sexual partners (especially for women) in South Africa, drought in Malawi with higher engagement in transactional sex, and industries of natural resource extraction and environmental degradation with epicenters of HIV transmission (4, 6). Recent modeling of changing temperatures and HIV prevalence across 25 sub-Sahara African countries predicts somewhere between 11.6 and 16 million additional acquisitions of HIV by 2050 due to findings connecting an increase in temperature, migration, and use of the sex industry (7). These connections underscore the need for specific attention to the overlapping effects of proximal and distal vulnerabilities perpetuated by the climate crisis and the HIV epidemic, which disproportionately affect populations marginalized by the climate crisis, exposure to HIV, or both.

Over the past decade, iterative frameworks have been developed that take slightly different approaches to understanding the climate crisis-HIV nexus: mapping the complex and multidirectional pathways connecting climate change impacts and potential exposure to HIV; illustrating the interconnectedness of location and vulnerability to the climate crisis and HIV; and establishing associations between the outcomes and determinants at play in the larger climate crisis-HIV relationship (1, 2). Despite these efforts to outline the connections between the climate crisis and HIV, few global actors in HIV prevention have linked the climate crisis and HIV in ways designed to improve prevention outcomes. Notably, the 2023 Country and Regional Operating Plan (COP/ROP) Guidance from the U.S. President's Emergency Plan for AIDS Relief (PEPFAR) includes a section on “Cross-Cutting Climate Attribution”, encouraging program planning to enhance resilience to the climate crisis, enable clean energy systems, and reduce greenhouse gas emissions through the promotion of sustainable land use practices (8). These activities are critical from an environmental perspective, but their potential impact on prevention is not mentioned.

HIV prevention efforts are critical to the UNAIDS goal of ending the AIDS epidemic by 2030. Moreover, the emergence of multiple biomedical prevention products and others in development mark an unprecedented and exciting era for HIV prevention. But even as new HIV prevention technologies become available, current and future prevention efforts will fall short if they do not consider the impact of the climate crisis on prevention outcomes. We propose four ways in which the linkages between the climate crisis and HIV can be translated into efforts to mitigate the impact of climate change on HIV prevention.

## Climate-responsive recommendations for HIV prevention and PrEP product introduction

By understanding the HIV-climate crisis nexus and the up- and downstream elements of that relationship, the HIV prevention product introduction ecosystem can better position its efforts to be more appropriately responsive. The following recommendations are meant to establish entry points for greater responsiveness. We have paired each of the four recommendations with an illustrative scenario and suggestions for operationalizing it. The scenarios are snapshots of the ongoing pressures inherent in the HIV-climate crisis relationship that demonstrate the need for intentional consideration of climate change impacts in HIV prevention and PrEP product introduction. The suggestions are not exhaustive but aim to lay groundwork upon which decisionmakers can build, prioritize, and make contextually appropriate adjustments. Table 1 captures the four recommendations, summarizes needs, and lists potential opportunities for operationalization and relevant actors responsible for implementing them. Given that the prevention product introduction landscape extends from planning and budgeting at a national scale to service delivery, uptake, and monitoring at the community and individual level, there are a multitude of actors who would benefit from a clearer grasp of how the climate crisis and HIV prevention interplay and impact HIV outcomes (9). Each recommendation may speak differently to various stakeholders and can be realized through collaboration and partnership. For example, policymakers can operationalize guidelines that leverage expanded evidence from researchers who directly engaged with end users on changing product needs and preferences due to climatic pressures.

**Table 1:** Summary of recommendations.

Recommendations	Situation	Gap	Opportunities	Relevant actors
Leverage existing and emerging research and lived experience to intentionally target and appropriately reach individuals affected by the climate crisis who may need or want PrEP.	Individual livelihoods are growing more fluid, by nature or force, and HIV programs must adapt to those dynamics.	Low awareness across the product introduction landscape of the climate crisis-induced factors that contribute to individual likelihood of exposure to HIV and overall incidence, and how that affects who may benefit from PrEP	Plan or advocate for: • Reevaluation of priority populations and who may benefit from PrEP • Expanded availability of PEP in climate-vulnerable settings • Differentiated service delivery strategies that support seasonal use of PrEP • Multimonth dispensing of PrEP • Addressing prevention needs in communities affected by natural disasters/extreme weather events	Donors, implementers, providers policymakers, end users
Emphasize the need for more climate-resilient PrEP products within the research and development pipeline.	The climate crisis continues to increase pressure on already weak healthcare systems, directly affecting PrEP access and method use.	Current PrEP methods do not map well to the instability created and sustained by the climate crisis.	Research and development efforts can be responsive by pursuing the expansion of a diverse PrEP method mix that: • Is user driven and not bound to clinic-based distribution • Requires less provider support for use • Has climate-resistant packaging • Requires less emissive supply chains • Limits environmental impact in waste management	Researchers, product manufacturers, donors, program implementers, supply-chain professionals, waste management policymakers, end users
Build a continued understanding of the role of the climate crisis-HIV relationship in product introduction through national collaboration.	Collaborative efforts between environmental and SRHR policymakers and program implementers are ongoing, and attention is growing.	Little-to-no dialogue and few tools specifically addressing climate-HIV product introduction efforts at the global or national level	Cross-sector relationship building and collaboration between policymakers in ministries of environment and health can support: • Climate-sensitive programs and infrastructure • Integrated climate-HIV or climate -SRH technical working groups • Coordination between HIV prevention and disaster response programs • National climate adaptation plans for communities with clear directives on HIV prevention as a part of the comprehensive SRH package and/or climate-sensitive HIV prevention implementation plans and guidelines • Strengthening existing integration of HIV and other SRH services	Policymakers in ministries of environment and health, donors, implementers, end users
Strengthen the integration of PrEP service delivery and response to intimate partner violence	Extreme weather events are increasingly common and more severe, contributing to economic and interpersonal stressors that result in increased incidence of IPV.	IPV tools and programming within HIV prevention do not consider the impact the climate crisis has on IPV outcomes.	Build out flexible, gender-sensitive, and climate-aware approaches to addressing IPV needs within HIV prevention by: • Strengthening systematic IPV counseling in PrEP service delivery, in particular at the community level • Establishing strong referral networks between IPV and prevention services in anticipation of climatic events	Program implementers, service providers, end users

### 1 Leverage existing and emerging research and lived experience to intentionally target and appropriately reach individuals affected by the climate crisis who may need or want PrEP

By nature or force, individuals and their needs are growing more mobile, and HIV prevention and PrEP product introduction programs must adapt to those dynamics (10). Programs and services that provide HIV prevention products must adopt the flexibility now required by communities most affected by climate change (11). Awareness of the climate crisis-induced factors that contribute to individual likelihood of exposure to HIV can better position programs to make PrEP available and build demand in anticipation of potential periods of increased transmission.

#### Scenario

Natural resource depletion in fishing communities caused by overfishing or seasonal algal blooms are now more frequent and severe due to warming temperatures, causing women to pursue transactional sex to supplement income lost by reduced fish stock (12–14).

The scenario shows how individual livelihoods are fluid and made more so by climate-induced changes. However, HIV product introduction planners such as policymakers, donors, and other implementing organizations, are not yet considering these populations made vulnerable by climate when determining whom to target and who may benefit from PrEP. In response, they can recognize that exposure to HIV does change with climatic influence — individuals' may benefit from post-exposure prophylaxis (PEP) more seasonally or may newly benefit from PrEP as their economic systems change because of climatic pressures. With this recognition, the product introduction and prevention stakeholders informing access to and availability of PrEP could tailor efforts more intentionally to increase coverage in a user-appropriate and climate-sensitive way.

### 2 Emphasize the need for more climate-resilient PrEP products within the research and development pipeline

Users' preferences and needs change dynamically during their PrEP use journeys, and the availability of methods that are responsive to users and enable informed choice is crucial to PrEP method uptake (11). The climate crisis continues to increase pressure on already weak healthcare systems, directly affecting how and when PrEP is (or is not) available. In response, the product pipeline must be responsive and still reach user needs even amidst increasing situations of instability or disrupted service delivery.

#### Scenario

Mobile communities and climate refugees have become more common as climatic events require relocation and cause displacement (4, 5). The timeframe for displacement as well as the presence of a robust healthcare system vary. A clinic may be washed away in climate-induced flooding, or a healthcare facility may be overburdened by climate refugees.

This scenario illustrates how, in the face of the climate crisis, healthcare systems cannot remain static and still meet client needs, but product development can balance this gap Flexibility is required for HIV prevention products to remain available, acceptable, and accessible. Research and development efforts can be responsive by pursuing and prioritizing the expansion of a diverse PrEP method mix within the product pipeline that reduces the burden on healthcare systems and centers individual users. This method mix can include both short- and longer-acting options that are less provider-dependent and minimize the footprint of supply chains or healthcare facilities, thus meeting the dynamic needs of individuals while simultaneously alleviating pressure on and cost to a healthcare system that may already be highly vulnerable to the effects of the climate crisis.

### 3 Build a continued understanding of the role of the climate crisis-HIV relationship in product introduction through national collaboration

Collaborative conversations about health and the climate crisis have begun at the global level, often under the expanding population, health, and environment (PHE) umbrella (15, 16). Sexual and reproductive health (SRH) and rights advocates have also begun to call for integrated programming that addresses the burden of climate crisis (17, 18). While the essential package of SRH services is inclusive of HIV, few of these conversations center HIV prevention or use a product introduction framing to understand outcomes. National-level dialogue and tools designed specifically for integrated climate-HIV efforts are another gap; conversations at this level have begun with advocates in the SRH space, but not within HIV prevention (19).

#### Scenario

Policymakers establish a coordinated mechanism that partners health, environment, and climate policies into national PHE guidance. This guidance brings together primary or integrated healthcare and biodiversity conservation or natural resource management in a coordinated manner but does not include HIV prevention or product introduction considerations.

Much remains unknown about the direct relationship between the HIV epidemic and the climate crisis. Yet just as inroads are continually made to integrate HIV and other health areas because of intertwined client needs, preferences, and outcomes, so too could policymakers, program developers, and all other relevant prevention actors forge integrated, cross-sector mechanisms that approach product introduction from both a climate and an HIV perspective. There is ample opportunity to draw from existing programmatic and policy-focused recommendations built within the broader SRH community, and hone interventions, policy, or service delivery for HIV prevention and product introduction where appropriate. Such mechanisms would not only facilitate climate-sensitive directives for HIV prevention practitioners and product introduction at large but would also create opportunities to better inform changes in public health infrastructure and policy in response to increasingly severe climate events.

### 4 Strengthen the integration of PrEP service delivery and response to intimate partner violence

Both the HIV epidemic and the climate crisis have gendered impacts, and the burden of each is not felt equitably around the world or within a given community but instead rests largely on women and adolescent girls (20). Notably, the relationship between climactic events and intimate partner violence (IPV) is recognized: climate-induced events or stress lead to rising rates of IPV (21).

#### Scenario

Natural disasters correspond with surging rates of IPV as stability around property, income, and social networks becomes strained (22). In a community living through cyclone season, individual stress—exacerbated by an approaching cyclone or subsequent damage, concerns about potential displacement, and fears of economic loss—leads to higher rates of IPV and increases the likelihood of HIV exposure for those experiencing IPV. Disaster relief or adaptation efforts have yet to employ approaches that address these interconnected outcomes.

PrEP programs must overlay the evidenced relationship between IPV and climatic events with existing knowledge that IPV is an indicator for HIV exposure. While addressing IPV is a recommended part of PrEP and integrated SRH delivery programs, IPV response is not always robust and systematically integrated. PrEP programs reaching communities affected by the climate crisis, and in particular severe climatic events, need to be particularly sensitive to the increased likelihood of IPV among potential PrEP users and ensure service delivery involves asking about IPV and responding appropriately to disclosures. Strengthening this crucial component of the PrEP service delivery package will better position HIV prevention efforts to respond to the role the climate crisis plays in IPV and therefore HIV outcomes.

## Discussion

The time-sensitive nature of the climate crisis and the ongoing, population-specific impacts of the HIV epidemic should encourage HIV prevention practitioners to act intentionally and urgently. We hope actors in the prevention field find our recommendations relevant and apply this roadmap to specific elements of prevention work, such as product introduction or service delivery. Notably, our recommendations also may apply to areas beyond HIV prevention, such as product introduction work in family planning or across the HIV care and treatment cascade. Further, the existing work found within the broader SRH space offers a critical foundation upon which HIV and product introduction programming can build and advance integration of essential services.

Optimizing the impact of prevention efforts requires taking the climate crisis into consideration and understanding its impact on HIV outcomes. Adapting or expanding upon our recommendations will be critical to best serve those most marginalized at the intersection of the climate crisis and HIV epidemic.

## Data Availability

The original contributions presented in the study are included in the article/Supplementary Material, further inquiries can be directed to the corresponding author.
